# Leukemia and Risk of Venous Thromboembolism: A Meta-analysis and Systematic Review of 144 Studies Comprising 162,126 Patients

**DOI:** 10.1038/s41598-017-01307-0

**Published:** 2017-04-26

**Authors:** Ying-Ying Wu, Liang Tang, Ming-Huan Wang

**Affiliations:** 1 Department of Oncology, Tongji Hospital, Huazhong University of Science and Technology, Wuhan, Hubei China; 2 Institute of Haematology, Union Hospital, Tongji Medical College, Huazhong University of Science and Technology, Wuhan, Hubei China; 3 Department of Neurology, Tongji Hospital, Tongji Medical College, Huazhong University of Science and Technology, Wuhan, Hubei China

## Abstract

Venous thromboembolism (VTE) has significant clinical implications in leukemia patients. However, the actual frequency of this complication remains unknown. We performed a systematic review and meta-analysis to better estimate the frequency of this complication and to assess the risk factors that contribute to its occurrence. We searched several databases, including PubMed, Embase, and Web of Science, and assessed study quality using the Newcastle–Ottawa scale. The pooled frequency of VTE in leukemia patients was calculated. A total of 144 studies met the eligibility criteria. The incidence rate (IR) of VTE from 72 prospective studies comprising 9,061 patients was 5% (95%CI: 4–6%). The incidence rate (IR) of VTE in ALL, CLL, total-AML, and CML population was 5% (95%CI: 4–6%), 3% (95%CI: 2–5%), 6% (95%CI: 4–8%) and 13% (95%CI: 1–36%). The incidence of VTE was markedly decreased among ALL patients who received anticoagulation treatment (IR: 1%, 95%CI: 0–6%) or concentrates therapy (IR: 3%, 95%CI: 0–9%). The overall incidence of VTE in the leukemia population was high, particularly in transplant recipients, who had the highest risk (IR: 8%, 95% CI: 4–13%). Prophylactic approaches could significantly decrease the occurrence of VTE events.

## Introduction

Cancer is associated with an increased frequency of venous thromboembolism (VTE)^1^. The incidence of thromboembolic events in cancer patients is approximately four to six-fold higher than in the general population^2^.

Recent studies have shown that patients with leukemia may have an incidence of thrombosis as high as (or even higher than) other cancer patients^3–6^. The reported incidence of VTE in leukemia varies from 1.1% to 36.7%^7^. This wide range in the reported incidence may be related to variations in the disease subtype and stage that were examined, the VTE definition (symptomatic *versus* asymptomatic) used, the diagnostic methods used, the study design (prospective *versus* retrospective analysis), and the leukemia treatment protocol. Thrombosis has a number of risk factors, including hereditary thrombophilia, the insertion of central venous catheters, exposure to a combination of steroids and asparaginase during induction, and the presence of hypercoagulation. In addition, cancer cells may alter hemostasis through direct interactions with endothelial cells to produce a prothrombotic environment.

In cancer patients, VTE results in the delay or withdrawal of chemotherapy, the second-highest cause of mortality and morbidity, a reduced quality of life, and a high cost burden. The American Society of Clinical Oncology (ASCO) originally developed guidelines for VTE in cancer patients, but further studies are urgently needed to define more precise risk stratifications^8^. Studies have indicated that VTE is more likely to occur in leukemia patients; however, these results are difficult to interpret because of a previous lack of effort to systematically evaluate research data from diverse sources characterizing the incidence of VTE among leukemia patients.

There is an urgent need to determine accurately the prevalence of thrombosis in leukemia patients, as this knowledge may be helpful in developing therapeutic or preventive strategies. The aims of the present meta-analysis were to determine the incidence of systematic VTE in leukemia, stratified based on risk factors, and to assess which patients are considered to be at a particularly high risk for VTE.

## Methods

### Data Source

This systematic review was implemented in accordance with the Preferred Reporting Items for Systematic Reviews and Meta-Analyses (PRISMA) statement^9^. We searched PubMed, Embase, and Web of Science to identify relevant studies. The search terms included “venous thromboembolism”, “deep venous thrombosis”, “pulmonary embolism”, and “leukemia”. Furthermore, citations were supplemented by cross checking the reference lists of eligible studies and relevant reviews to identify additional published data.

### Study selection and data extraction

All studies that reported associations between VTE risk and leukemia were included in this review, with no restrictions on publication date, region, stage of disease, or status. Studies that included patients with baseline VTE or that reported superficial phlebitis, or thrombophlebitis but not VTE were excluded. Two investigators independently and separately performed the data extraction according to PRISMA guidelines. The information extracted from each study included the first author, year of publication, country, study design, type of leukemia examined, research purpose, number and type of catheters placed, and number of transplant recipients included. The incidence VTE data in each study was extracted for the following meta-analysis. A third investigator resolved any discrepancies.

### Subgroup analysis

The incidence rate of VTE was separately estimated for different subgroups of studies according to study design, type of leukemia examined, patients’ age, patients’ geographic location, insertion of CVCs (central venous catheters) or PICCs (peripherally inserted central catheters), thromboprophylaxis, receipt of transplantation, and year of study publication.

#### Study design

Two distinct IRs for VTE were determined based on study design, one for prospective studies and one for retrospective studies.

#### Type of leukemia

The studies were subgrouped according to the leukemia subtypes of ALL (acute lymphoblastic leukemia), CLL (chronic lymphoblastic leukemia), AML (acute myeloid leukemia), APL (acute promyelocytic leukemia), and CML (chronic myeloid leukemia).

#### Age of patients

ALL is one of the most prevalent cancers in pediatric patients, and VTE is a common complication in the course of the disease. A scarcity of studies describing VTE in adult patients with ALL was observed. The occurrence of VTE in adulthood was primarily recorded in patients with the CLL, AML, APL, or CML subtype. Thus, we only sorted study participants with the ALL subtype into older or younger than18 years of age.

#### Insertion of CVC or PICC

Studies assessing the effect of CVC or PICC insertion were included in a separate meta-analysis to estimate the risk of VTE in leukemia patients with catheter implantation.

#### Thromboprophylaxis

Thromboprophylaxis was defined as the use of supplement therapy (antithrombin concentration or fresh frozen plasma) or anticoagulation therapy (unfractionated heparin, low-molecular-weight heparin, aspirin, or warfarin) within a particular ALL population. A total of 17 studies included ALL leukemia patients receiving thromboprophylaxis, and these were divided into supplement therapy, anticoagulation therapy, and combined therapy subgroups to evaluate the beneficial effect of thromboprophylaxis.

#### Transplantation

Five studies comprising more than half of all leukemia-mandated transplantations we analyzed were included in a separate meta-analysis to calculate the risk of VTE in the leukemia patient cohort who underwent transplantation.

#### Year of study publication

The studies were subgrouped based on publication dates between 1990 and 1999, 2000 and 2009, and 2010 and 2014.

### Data synthesis and analysis

Incidence rate was defined as the number of people who developed the DVT over a specified time period following diagnosis/treatment. The pooled incidence rate of VTE, 95% confidence intervals, and *P* values were calculated using the random effects method of DerSimonian and Laird^10^. Heterogeneity, the variation between study results, was quantified using the *I* ^2^ statistic^11^. Subsequent subgroup analyses were performed to explore the between-study heterogeneity. We classified heterogeneity as low, moderate, or high based on an *I* ^2^ statistic of 25%, 50%, or 75%, respectively, according to Higgins and colleagues^11^. Publication bias was assessed using Egger’s regression test. All tests were 2-sided, and statistical significance was defined as *P* ≤ 0.05. We performed several sensitivity or influence analyses to test the robustness of these findings. Analyses were performed using R statistical software with the metaphor package.

## Results

**Figure 1:**
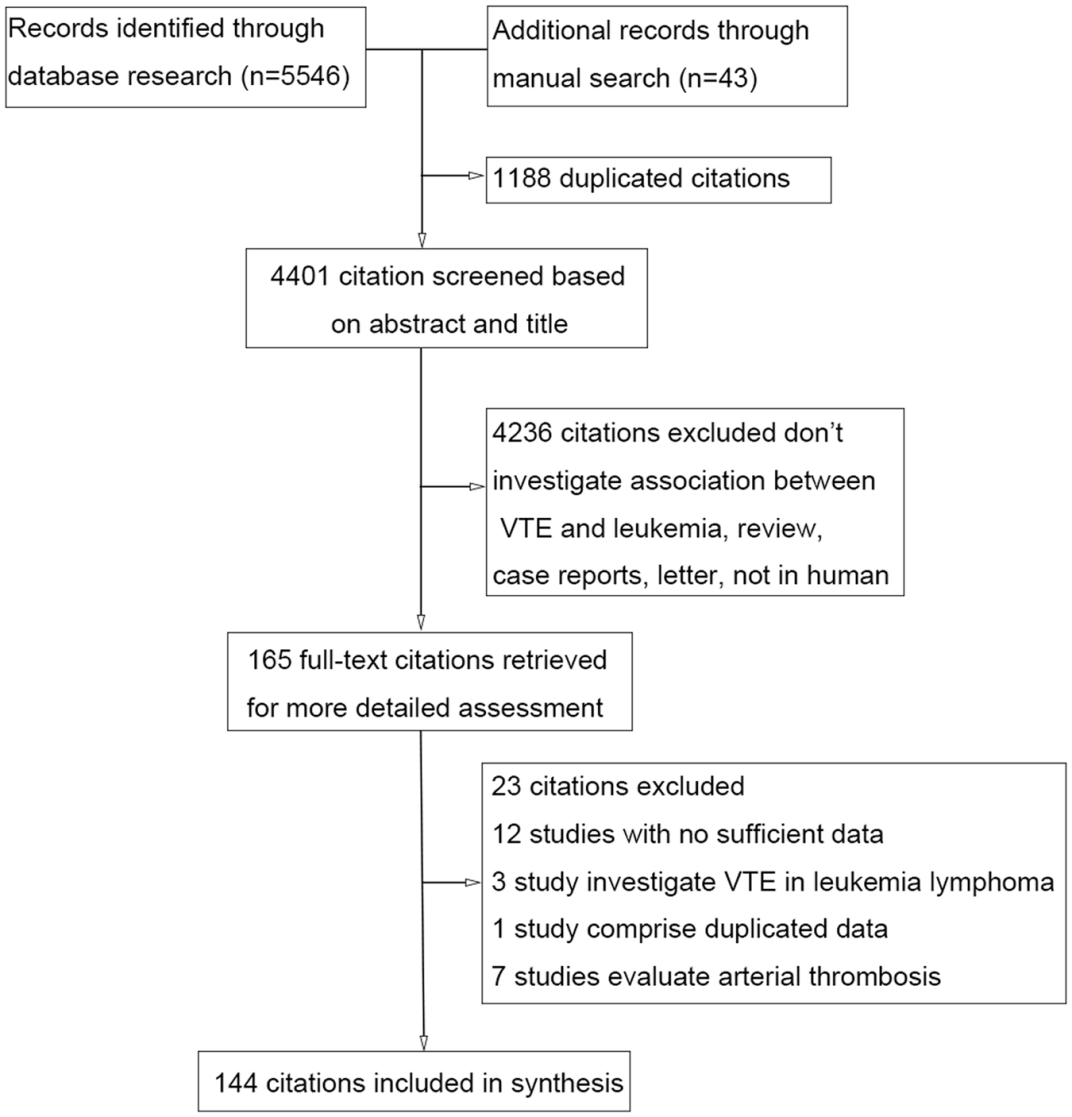
Flowchart of study selection method.

A total of 5,589 potentially relevant articles were identified using our search strategy. After screening the title and abstract and subsequently reviewing the full text, we included 144 articles that fulfilled the criteria in this study. All these studies were full-length reports published in peer-reviewed journals; except for 5 articles presented as letters. Figure 1 shows a flowchart of the study selection process.

**Figure 2:**
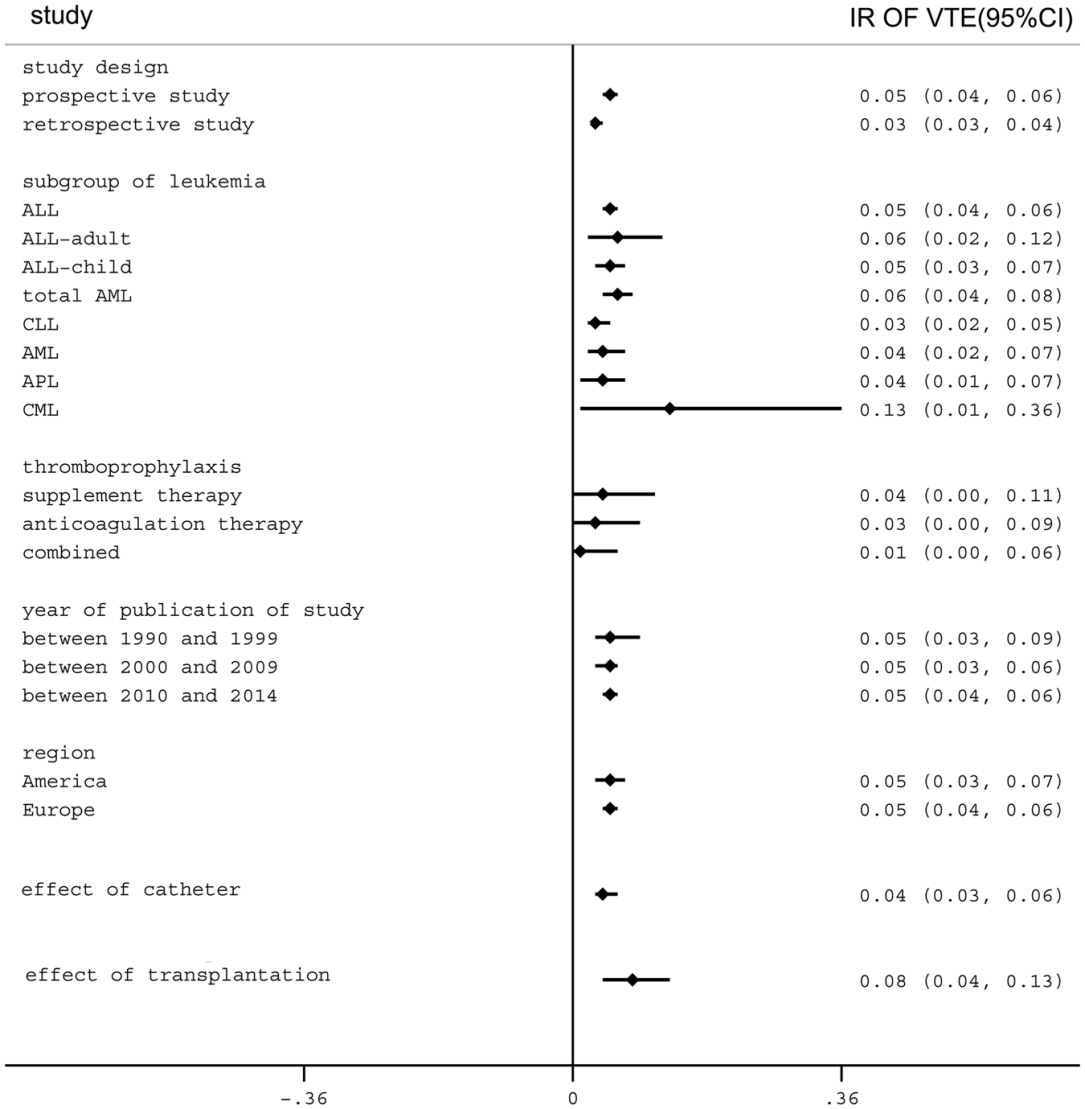
Summary of the categorical meta-analysis. IR = incidence rate. VTE = venous thromboembolism.

Eligible studies varied in subject number, ranging from 12 to 47,234 patients. Among the 144 studies selected, there were 69 retrospective studies, 72 prospective studies, and 3 studies that contained both retrospective and prospective analysis. Details of the included studies are summarized in Table S1. Among the prospective cohort studies, 28 reports were obtained from America, 44 reports were obtained from Europe, 1 report was obtained from Asia, and 1 study was conducted in Africa. Subgroup analysis was performed to identify groups at high risk for VTE within leukemia populations. The results of the subgroup analysis are shown in Fig. 2. There was no evidence of publication bias.

### IRs

The included articles were divided as described in Table 1. Considering all prospective studies (72 studies, 75 individual cohorts, 9,061 patients, and 448 venous thrombosis events), the incidence rate of VTE was 5% (95%CI, 4–6%). Nevertheless, when the analysis was restricted to retrospective studies, a substantially decreased risk was observed (3%, 95%CI, 3–4%). Because memory recall of events in retrospective studies can be erratic, prospective design is the best method to observe the frequency of a phenomenon; therefore, only prospective studies were considered for subsequent analyses.

**Table 1:** Classification of 144 selected studies according to primary research objectives and methodological design.

Class of study	No. of studies	No. of cohorts	Patients	Events	Incidence of VTE (95% CI)
A	75	87	9,061	448	0.05 (0.04, 0.06)
B	40	41	2,431	108	0.06 (0.04, 0.08)
C	20	21	5,308	263	0.04 (0.03, 0.05)
D	7	7	308	23	0.06 (0.02, 0.14)
E	72	71	153,062	2,770	0.03 (0.03, 0.04)

A indicates all prospective studies comprising B, C, D. B indicates prospective studies evaluating the outcomes of clinical therapeutic protocols describing VTE as a complication. C indicates prospective studies investigating the prevalence of clinical venous thrombotic events. D indicates prospective studies evaluating laboratory parameters of hypercoagulability thatdescribe clinical thrombotic complications as additional information. E indicates retrospective studies.

### Leukemia subtype

**Figure 3:**
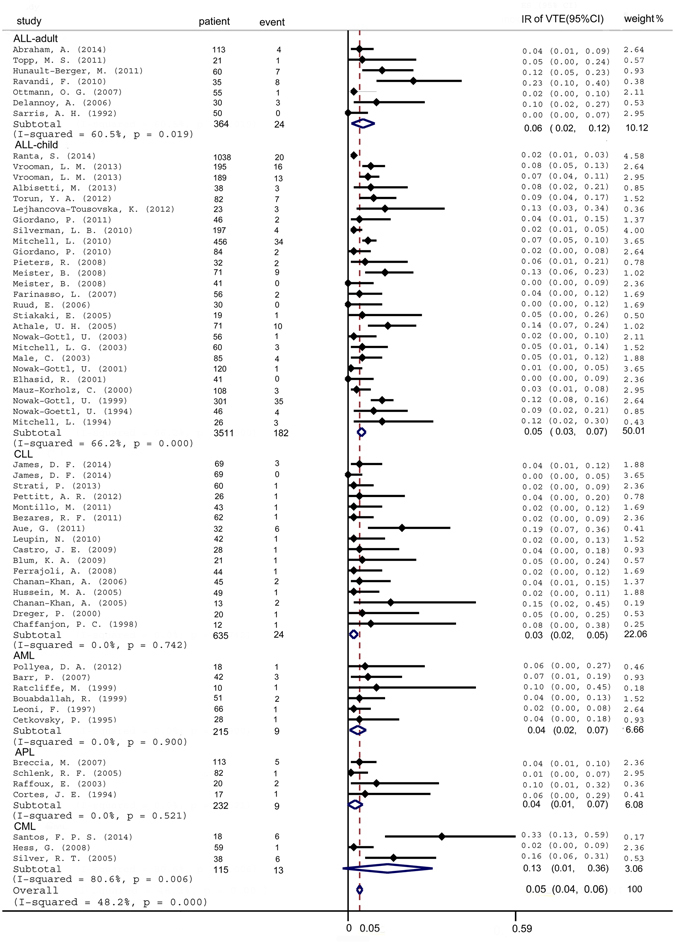
Forest plot of the risk of VTE in participants stratified by leukemia subtype. Random effects meta-analysis showing individual and pooled weighted incidence of leukemia-related VTE in studies, stratified by ALL-adult, ALL-child, CLL, AML, APL, and CML. ALL = acute lymphoblastic leukemia. CLL = chronic lymphoblastic leukemia. AML = acute myeloid leukemia. CML = chronic myeloid leukemia. APL = acute promyelocytic leukemia. IR = incidence rate. VTE = venous thromboembolism.

Given the potentially different risk of patients with distinct subtypes of leukemia, an exploratory analysis of patients stratified by leukemia subtype was performed. Comparisons between ALL-adult, ALL-child, CLL, AML (except APL), total-AML, APL, and CML showed differences in the occurrence of VTE. Notably, compared with other subtypes, CML patients had the highest incidence (Fig. 3).

### Influence of venous thromboembolism prophylaxis

**Figure 4:**
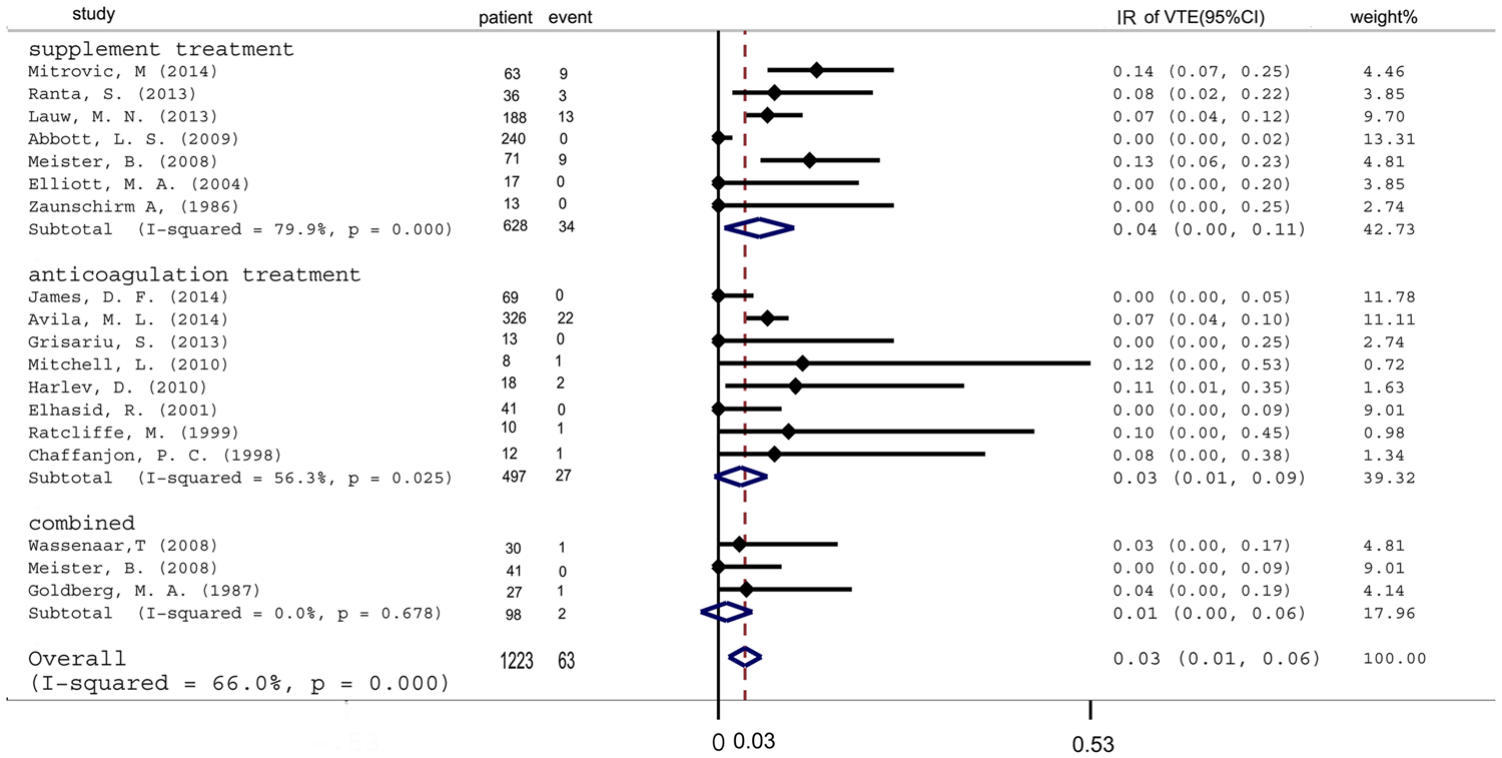
Pooled incidence of venous thromboembolism after receiving prophylaxis. IR = incidence rate. VTE = venous thromboembolism.

VTE prophylaxis consisted of replacement or anticoagulation therapy. The incidence of VTE was markedly decreased among ALL leukemia patients receiving anticoagulation treatment (IR: 1%, 95%CI: 0–6%) and supplement therapy, although to a lesser degree (IR: 3%, 95%CI: 0–9%) (Fig. 4).

### Presence of CVC or PICC

**Figure 5:**
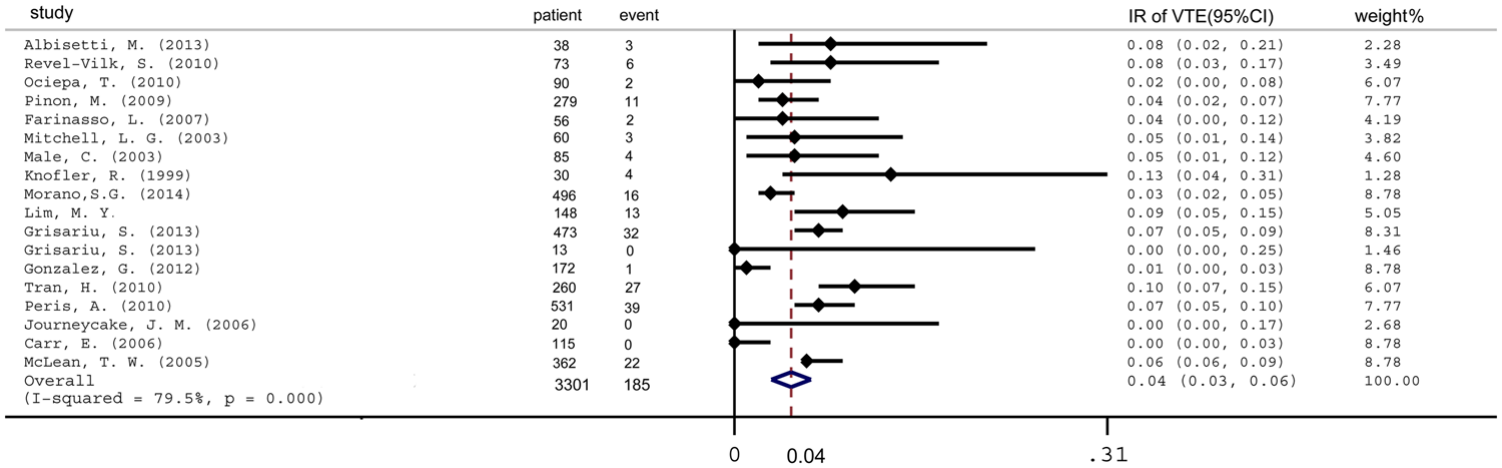
Pooled incidence of venous thromboembolism in patients with a catheter. IR = incidence rate. VTE = venous thromboembolism.

The presence of a catheter is closely associated with episodes of VTE. A total of 185 events were reported in 3,301 patients with catheter implantation. However, the pooled estimate of 18 studies showed no association between catheter placement and an increased risk of developing VTE (IR: 4%, 95%CI: 3–6%) (Fig. 5).

### Influence of transplantation

**Figure 6:**
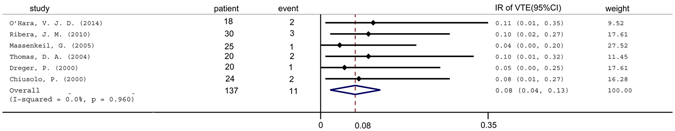
Pooled incidence of venous thromboembolism in transplant recipients. IR = incidence rate. VTE = venous thromboembolism.

Transplantation (allogeneic hematopoietic cell transplantation or autogenic hematopoietic cell transplantation) was administered to either more than half or all patients in 6 of the selected studies. A meta-analysis of these 6 studies indicated that transplant recipients had the highest incidence of VTE (IR: 8%, 95%CI: 4–13%) (Fig. 6).

### Effect of publication period

**Figure 7:**
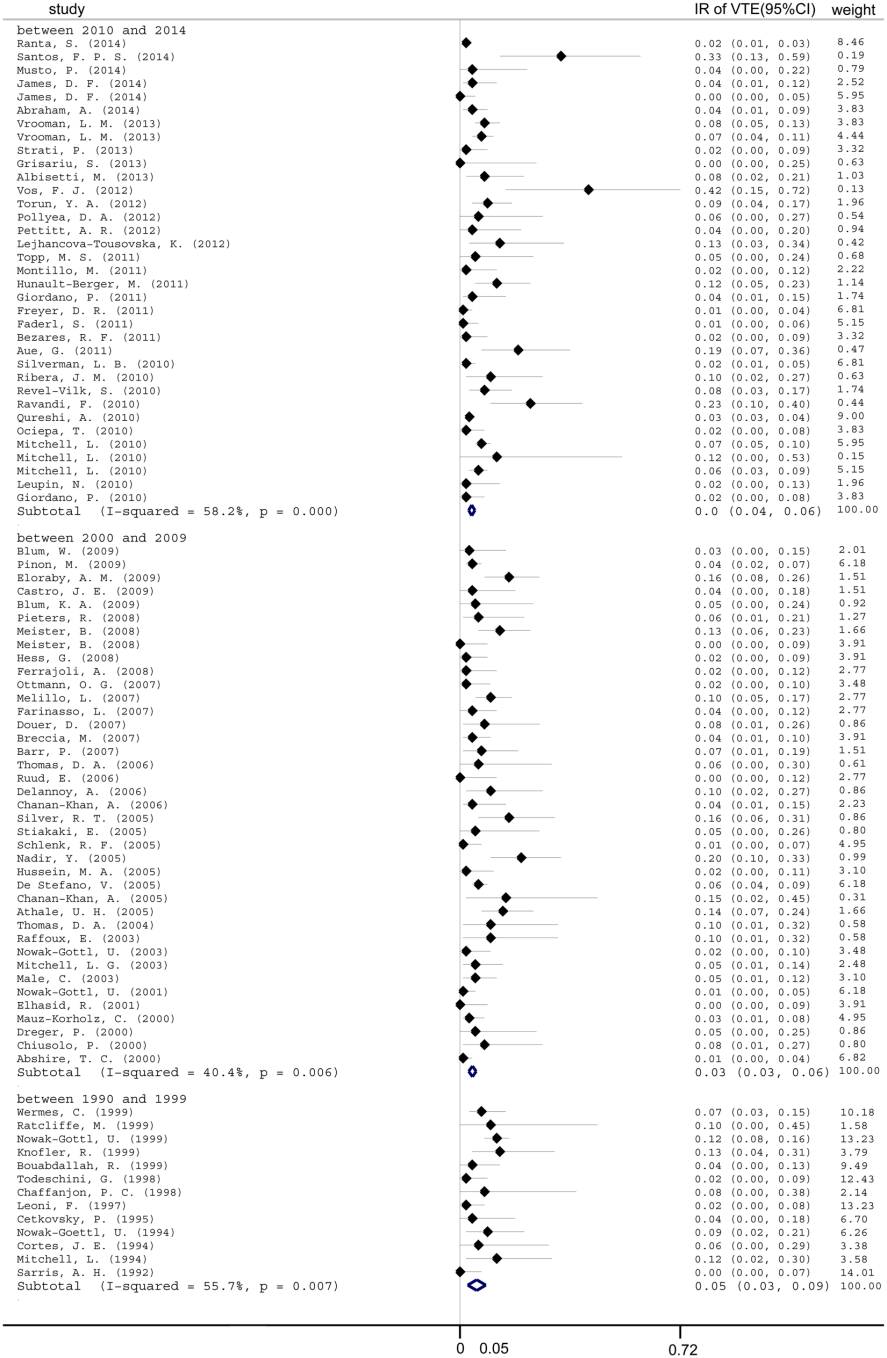
Pooled incidence of venous thromboembolism subgrouped according to publication year. IR = incidence rate. VTE = venous thromboembolism.

We hypothesized that the incidence of VTE observed in leukemia patients may have increased over the past few decades due to more aggressive chemotherapy treatment and more accurate diagnostic methods. Therefore, we investigated the impact of publication period on the incidence of symptomatic VTE in the leukemia patient population. Unexpectedly, leukemia patients had a similar magnitude risk regardless of the publication period: 5% (95%CI, 4–6%) between 2010 and 2014, 5% (95%CI, 3–6%) between 2000 and 2009, and 5% (95%CI, 3–9%) between 1990 and 1999 (Fig. 7).

## Discussion

VTE is a common complication of malignant cancer^12^. The annual rate of VTE in cancer patients is approximately 1 in 200, while the annual incidence of a first episode of deep venous thrombosis or pulmonary embolism in the general population is 117 in 100,000^13^. While solid tumors have long been associated with an increased risk of VTE complications, recent studies^14^ have suggested that the rate of VTE in patients with hematologic malignancies may also be potentially high. However, the precise incidence and risk of VTE in leukemia was previously unknown.

In the present meta-analysis, we determined that the overall incidence of VTE in leukemia is approximately 5%. Among all leukemia subtypes, CML patients have the highest rate of VTE.

### Incidence

Cancer is associated with venous thrombosis. Moreover, the rate of cancer-associated VTE increased by 28% in more than one million cancer patients from 1995 to 2003^15^. The overall risk of VTE increased 7-fold in patients with malignancy compared with those without malignancy^16^.

Previous studies have reported that thrombosis is the second leading cause of death in cancer patients^17, 18^. In one retrospective study, an association between VTE and a subsequent diagnosis of cancer was observed. This risk was substantially elevated during the first six months of follow-up following the thrombotic event and rapidly declined to a constant level slightly above 1.0% after one year^18^.

Notably, venous thrombosis was more frequent than clinically observed, remaining undetected in many cases. Significantly, venous thrombosis prophylaxis could decrease the risk of the occurrence of VTE; however, some studies have reported an adverse effect of prophylaxis. Furthermore, non-pharmacological methods, such as the prevention of infection, may be useful in preventing VTE. Consequently, large, randomized, controlled studies that assess both the risks and benefits associated with prophylaxis therapy are urgently needed.

### Transplantation

VTE is a major complication after transplantation. The impairment of fibrinolysis from corticosteroid use, the pro-coagulant effects of immunosuppressive therapy, and the endothelial damage due to cytomegalovirus infection all predispose recipients to developing VTE; thus, the rate of transplantation-associated VTE is high. The incidence of VTE after renal transplantation ranged from 1.5% to 8.3%^19–21^. VTE after liver transplantation has been described as a complication of this procedure with an incidence between 3.7% and 4.6%^22–24^.

### Effect of publication period

A progressively increasing incidence of leukemia-associated VTE was not observed, even though more aggressive chemotherapies and more accurate diagnosis tools have been implemented in recent years. Improved healthcare systems may be potential reasons for this observation. However the association between study period and VTE incidence requires further investigation.

### Thromboprophylaxis

ASCO, the National Comprehensive Cancer Network (NCCN), and other professional organizations have published guidelines for VTE prophylaxis and treatment in patients with cancer^25^. The prevention of VTE during treatment is complex, as thrombosis and bleeding need to be balanced. Different measures to prevent VTE have been described, such as supplementation with fresh frozen plasma (FFP) or antithrombin concentrate and the prophylactic use of anticoagulants, including low-molecular-weight heparin (LMWH). The results of supplementation therapy have been contradictory. Several studies showed no significant decrease in VTE occurrence^26, 27^; however, Lauw showed that VTE incidence was significantly lower with FFP supplementation than with no FFP^3^. Pilot studies assessing the prophylactic use of anticoagulants showed a reduction in the incidence of VTE without major bleeding^28, 29^. These findings provide evidence for the usefulness of VTE prevention in leukemia populations with a high risk of thrombosis.

### Presence of catheters

Catheters may significantly contribute to the occurrence of VTE. However, in our manuscript the contribution of catheters to thrombosis in leukemia patients has not been thoroughly investigated and is likely underestimated in the present meta-analysis because only a few of the selected studies reported catheter use. The incidence of symptomatic catheter-related thrombosis in patients with malignancies ranged between 11% and 45% in studies of cancer populations with catheters^30^, with an even higher rate of asymptomatic VTE^31^. These findings suggest that catheters are an important risk factor contributing to the occurrence of venous thrombosis.

### Impact of region

No difference was observed in the risk of thrombosis in Europe and America. We only discovered 1 report obtained from Asia, and 1 study conducted in Africa, so we can’t compare the VTE risk in Asian or African leukemia population with Europen or American leukemia population. Some lines of evidence indicated the VTE incidence was highest in individuals of African ancestry, followed by Caucasians, and lowest in Asions.

The pathogenesis of VTE in leukemia is complex and multifactorial and can be attributed to the disease itself, chemotherapy use, and other acquired risk factors. Truelove *et al*. determined that asparaginase decreased the levels of anticoagulation factors and proteins, particularly antithrombin, and upregulated tissue factors via the activation of white cells and endothelium, resulting in thrombin initiation in ALL patients. Steroids contribute to a hypercoagulated state through the elevation of the VIII/von Willebr and factor complex and a hypofibrinolytic state through the increase of plasminogen activator inhibitor^32^.

The results of the present study should be considered in the context of several limitations. First, most of the included studies did not have a comparator group, reducing the ability to generate pooled relative risks for VTE. Second, almost half of the prospective studies were randomized clinical trials, in which the enrolled patients must meet rigorous criteria that exclude many patients at high risk. This limitation may result in an underestimation of VTE incidence in leukemia. Third, data for VTE prophylaxis were scarce, limiting our understanding of the benefit vs. risk profile of VTE prophylaxis.

Nonetheless, the present study has notable strengths. First, we included all subtypes of leukemia; to the best of our knowledge, the present study was the largest and most comprehensive review of VTE incidence associated with leukemia. Second, we addressed the hypothesis that transplantation is a risk factor for developing VTE. Consequently, these findings may provide education to holistic clinicians, researchers, and policy makers concerning the incidence, risk factors, and management of VTE in leukemia patients. Third, the ultimate aim of this review was to provide a basis for prospective studies in leukemia patients to facilitate the establishment of guidelines for the identification of high-risk groups, prophylaxis, and the management of thrombotic complications in patients with cancer.

## Electronic supplementary material

Table S1
