# Supplementary material for: Leukemia and Risk of Venous Thromboembolism: A Meta-analysis and Systematic Review of 144 Studies Comprising 162,126 Patients
Source: Sci Rep. 2017 Apr 26;7:1167. doi: 10.1038/s41598-017-01307-0 (PMC5430898; doi:10.1038/s41598-017-01307-0)
Supplement: Supplementary file 1 — Table S1 [file 41598_2017_1307_MOESM1_ESM.pdf]

Leukemia and Risk of Venous Thromboembolism: A Meta-analysis and Systematic Review of 144 studies comprising 162126 patients

Ying-Ying Wu[1,*]; Liang Tang[2,*]; Ming-Huan Wang[3];

[1]Department of Oncology, Tongji Hospital, Huazhong University of Science and Technology, Wuhan, Hubei, China;

[2]Institute of Haematology, Union Hospital, Tongji Medical College, Huazhong University of Science and Technology, Wuhan, Hubei, China;

[3]Department of Neurology,Tongji Hospital, Tongji Medical College, Huazhong University of Science and Technology, Wuhan, Hubei, China;

Ying-Ying Wu and Liang Tang contributed equally to this work.

Correspondence to: Ming-Huan Wang

Department of Neurology,Tongji Hospital, Tongji Medical College, Huazhong University of Science and Technology, Wuhan, 430030, China;

E-mail:jonyaaa4@163.com

Table S1.characters of included studies

	Region	Study design	Patient population	Research purpose	Total patients	VTE event (n[% of event/tatal patient])	VTE prophylaxis	Insertion Of PICC or CVL	transplantation
Lee,Y.G. (2015) [1]	Korea	RC	AML	Clinical Thrombotic Event	811	26	NA	13catheter-related VTE	351Hematopoietic stem.cell.transplantation 307Allogeneic 44Autologous
Ranta,S. (2014)[2]	Sweden	PC	ALL	Incidence of Cerebral sinus venous thrombosIs	1038	20	NA	NA	NA
Santos,F.P.S. (2014)[3]	USA	PC	CML-CP	Clinical Thrombotic Event in Splenectomy	18	6	NA	NA	NA
O'Hara, V. J. D. (2014)[4]	USA	RC	chronic leukemia	Clinical Thrombotic Event in HCT	18	2	NA	1 line associated VTE	All recievean autologous or allogeneic HCT
Musto, P. (2014)[5]	Italy	PC	PPCL	Clinical trials	23	1	NA	NA	NA
Morano,S.G. (2014)[6]	Italy	RC	Leukemia	Evaluation of complication of catheter	496	16	NA	All patients insertion with CVC	NA
Mitrovic, M. (2014)[7]	Serbia.	RC	APL	Clinical Thrombotic event	63	9	Partial administered with FFP	3 CVC associated VTE	NA
James, D. F. (2014)[8]	USA	PC	CLL	Clinical trials	69<br>69(prophylaxis)	3<br>0	 Asipilin	NA	NA
Bach, A. G. (2014)[9]	Germany	RC	Leukemia	Clinical Thrombotic event	75	1	NA	NA	NA
Avila, M. L. (2014)[10]	Canada	RC	ALL	Assessment Of anticoagulation management	326	22	22 VTE with LMWH prophylaxis	9Line-related DVT	NA
Abraham, A. (2014)[11]	India	PC	T-ALL	Clinical trials	113	4	NA	NA	NA
Walker, A. J. (2013)[12]	UK	RC	Leukemia	Clinical Thrombotic event	2057	51	NA	NA	NA
Vrooman, L. M. (2013)[13]	USA	PC	ALL	Clinical trials	195<br>189	16<br>13	NA	NA	Partial patients receive HCT
Strati, P. (2013)[14]	USA	PC	CLL	Clinical trials	60	1	NA	NA	NA

Santoro, N. (2013)[15]	Italy	RC	ALL	Clinical Thrombotic event	2042	48	No thromboprophylaxis	1408 has a CVL<br>24 have a CVL related VTE	NA
Ranta, S. (2013)[16]	Sweden	RC	ALL	Effect of thromboprophyl-axis	39<br>10<br>36(prophylaxis)	0<br>2<br>3	23/36 has AT3 substitution regimen	All patients insertion with CVC	NA
Lim, M. Y. (2013)[17]	USA	RC	AML	Evaluationof complicationof catheter	148	13	NA	64Hickman catheter, 84 PICC	NA
Lauw, M. N. (2013)[18]	The Netherlands	RC	ALL	Effect of thromboprophylaxis	188(prophylaxis)<br>43	13<br>11	FFP	12/24 has CVC related VTE	YES
Guzman-Uribe, P. (2013)[19]	Mexico	RC	Acute leukemia	Clinical Thrombotic event	442	15	NA	8/15 has CVC related VTE	NA
Grisariu, S. (2013)[20]	Israel	RC<br>PC	Acute leukemia	Evaluationof complicationof cathet	473<br>13	32<br>0	no prophylaxis<br>LMWH treatment	447PICC<br>22Hickman<br>1 Port-a-Cath	162 transplantation
Chang, H. (2013)[21]	Taiwan	RC	APL	Clinical Thrombotic event	127	10	NA	5 has catheter related thrombosis	NA
Albisetti, M. (2013)[22]	Switzerland	PC	ALL	Evaluationof complicationof catheter	38	3	NA	All patients insertion with Port-A-Cath	NA
Vos, F. J. (2012)[23]	The Netherlands	PC	Leukemia	Clinical Thrombotic event	12	5	NA	Partial CVL	YES
Torun, Y. A. (2012)[24]	Turkey	PC	ALL	Assessment of inherited prothrombotic risk fators	82	7	No receiving heparin prophylaxis	35/82 inserted with CVL	NA
Thyagu, S. (2012)[25]	Canada	RC	Ph+ALL	Clinical trials	32	3	NA	NA	16 HCT
Rollig, C. (2012)[26]	Germany	RC	AML	Clinical trials	32	1	NA	NA	NA
Pollyea, D. A. (2012)[27]	USA	PC	AML	Clinical trials	18	1	NA	NA	NA
Pettitt, A. R. (2012)[28]	UK	PC	CLL	Clinical trials	26	1	NA	NA	Pantrtial
Lejhancova-Tousovska, K. (2012)[29]	Czech Republic	PC	ALL	Evaluate of Hypocoagulation state	23	3	No thromboprophylaxis	ALL 3 CVC related VTE	NA
Gonzalez, G. (2012)[30]	Tennessee	RC	ALL	Evaluationof complicationof catheter	172	1	No thromboprophylaxis	All patients insertion with CVC	NA
Akin, D. F. (2012)[31]	Turkey	RC	Acute leukemia	Assessment of inherited prothrombotic risk fators	135	2	NA	NA	NA
Whittle, A. M. (2011)[32]	UK	RC	CLL	Clinical Thrombotic event	268	14	NA	NA	NA
Topp, M. S. (2011)[33]	Germany	PC	B-ALL	Clinical trials	21	1	NA	1 Catheter thrombosis	Pantrtial
Santos, F. P. (2011)[34]	USA	RC	CML	Effect of erythropoietic-stimulating agents	608	10	NA	NA	NA
O'Brien, S. H. (2011)[35]	USA	RC	Leukemia	Clinical Thrombotic event	2,721	197	NA	NA	NA
Montillo, M. (2011)[36]	Italy	PC	B-CLL	Clinical trials	43	1	NA	NA	NA

Lipay, Natalie V. (2011)[37]	Republic of Belarus	RC	Leukemia	Clinical Thrombotic event	669	27	NA	Partial CVL	NA
Latagliata, R. (2011)[38]	Italy	RC	CML	Clinical trials	108	1	NA	NA	NA
Hunault-Berger, M. (2011)[39]	France	PC	Philadelphia chromosome-negative or BCR-ABL-negative ALL	Clinical trials	60	7	NA	All patients insertion with CVC	NA
Grace, R. F. (2011)[40]	USA	RC	ALL	Effect of asparaginase related thrombosis	548	48	NA	17 CVL-related VTE	NA
Giordano, P. (2011)[41]	Italy	PC	ALL	Clinical Thrombotic event	46	2	NA	NA	NA
Freyer, D. R. (2011)[42]	USA	PC	ALL	Clinical trials	162	2	NA	NA	NA
Faderl, S. (2011)[43]	USA	PC	ALL	Clinical trials	90	1	NA	NA	28 stem cell transplantation
Bezares, R. F. (2011)[44]	Argentina.	PC	CLL	Clinical trials	62	1	NA	NA	NA
Aue, G. (2011)[45]	USA	PC	CLL	Effect of lenalidomide to venous thrombosis	32	6	No-hrombotprophylaxis(except3 with a history of DVT has therapeutic anticoagulation)	No CVC related thrombosis	NA
Al-Aridi, C. (2011)[46]	Lebanon	RC	ALL	Clinical Thrombotic event	111	9	No thromboprophylaxis	All patients insertion with port-a-cath	NA
Visani, G. (2010)[47]	Italy	RC	CML	Clinical trials	114	1	NA	NA	4 allogeneic stemcell transplantation
Tran, H. (2010)[48]	USA	RC	Leukemia	Evaluationof complicationof catheter	260	27	No thromboprophylaxis	All patients insertion with PICC	NA
Silverman, L. B. (2010)[49]	USA	PC	ALL	Clinical trials of PEG-asparaginase	197	4	NA	NA	NA
Ribera, J. M. (2010)[50]	Spain	PC	Ph+ALL	Clinical trials	30	3	NA	NA	21SCT(allogeneic, 16; autologous, 5
Revel-Vilk, S. (2010)[51]	Israel	PC	ALL	Evaluationof complicationof catheter	73	6	No thromboprophylaxis	All patients insertion with CVC	NA
Ravandi, F. (2010)[52]	USA	PC	Ph+ALL	Clinical trials	35	8	NA	NA	partial
Qureshi, A. (2010)[53]	UK	PC	ALL	Effect of Asparaginase	1824	61	NA	29 CVL related thrombosis	NA
Peris, A. (2010)[54]	Italy	RC	Leukemia	Evaluationof complicationof cathete	531	39	NA	All patients insertion with CVC	NA
Ociepa, T. (2010)[55]	Poland	PC	Acute leukemia	Evaluation of complication of catheter	90	2	NA	All patients inserted with tunneled central lines	NA
Mitchell, L. (2010)[56]	Europe	PC	ALL	Clinical Thrombotic event	456<br>339<br>8	34<br>19<br>1(prophylaxis)	LMWH	7 CVL related VTE	NA
Leupin, N. (2010)[57]	Switzerland	PC	B-CLL	Clinical trials	42	1	NA	NA	NA
Harlev, D. (2010)[58]	Israel	RC	ALL	Effect of thromboprophyl-axis	62<br>18(FV, FII)	3<br>2(prophylaxis)	LMWH	1 PICC and CVL related VTE	NA
Giordano, P. (2010)[59]	Italy	PC	ALL	Effect of hemostatic alteration	84	4	NA	63/84CVC implanted	NA

Storring, J. M. (2009)[60]	Canada	RC	BCR-ABL negative ALL	Clinical trials	85	16	NA	3 CVL related VTE	12BMT
Blum, W. (2010)[61]	USA	PC	Acute leukemia	Clinical trials	35	1	NA	1 Catheter-associated DVT	8 BMT<br>1 Autologous<br>7 Allogeneic
Pinon, M. (2009)[62]	Italy	PC	ALL	Evaluation of complication of catheter	279	11	NA	All patients inserted with CVC<br>11CVC-thrombosis	NA
Ku, G. H. (2009)[63]	USA	RC	Acute leukemia	Clinical Thrombotic event	7876	287	NA,	Catheter VS no-catheter in AML HR 1.6,in ALL 2.2	NA
Eloraby, A. M. (2009)[64]	Egypt	PC	Acute leukemia	Effect of L-asparaginase	70	11	NA	NA	NA
Castro, J. E. (2009)[65]	USA	PC	CLL	Clinical trials	28	1	NA	NA	NA
Blum, K. A. (2009)[66]	USA	PC	CLL	Clinical trials	21	1	NA	NA	NA
Abbott, L. S. (2009)[67]	Canada	RC	ALL	Effect of thromboprophyl-axis	240<br>479(no-prophylaxis)	0<br>7	FFP or CYR supplement treatment	NA	NA
Wassenaar, T. (2008)[68]	USA	RC	APL	Effect of hemostatic alteration	30	1	Comprehensive Coagulopathy strategy	NA	NA
Pieters, R. (2008)[69]	The Netherlands	PC	ALL	Effect of asparaginase	32	2	NA	All patients inserted with port-a-cath	NA
Meister, B. (2008)[70]	Austria	PC	ALL	Effect of thromboprophyl-axis	71<br>41	9<br>0	with AT<br>with AT & LMWH	NA	NA
Hess, G. (2008)[71]	Germany	PC	CML	Clinical trials	59	1	NA	NA	5 SCT
Ferrajoli, A. (2008)[72]	USA	PC	CLL	Clinical trials	44	1	NA	NA	NA
Ottmann, O. G. (2007)[73]	Germany	PC	Ph+ALL	Clinical trials	55	1	NA	NA	NA
Melillo, L. (2007)[74]	Italy	PC	Acute leukemia	Clinical Thrombotic event	104	10	NA	1 CVC related VTE	NA
Farinasso, L. (2007)[75]	Italy	PC	ALL	Evaluation of complication of catheter	56	2	NA	2 CVC related VTE	NA
Khorana, A. A. (2007)[76]	USA	RC	Leukemia	Clinical Thrombotic event	46，977	197	NA	NA	NA
Douer, D. (2007)[77]	USA	PC	ALL	Effect of asparaginase	25	2	NA	NA	NA
Breccia, M. (2007)[78]	Italy	PC<br>RC	APL	Clinical Thrombotic event	113<br>80	5<br>1	NA	NA	NA
Barr, P. (2007)[79]	USA	PC	AML	Clinical trials	42	3	NA	None CVC-related VTE	PARTIAL
Thomas, D. A. (2006)[80]	USA	PC	ALL	Clinical trials	16	1	NA	NA	NO
Sakuma, M. (2006)[81]	Japan	RC	Leukemia	Clinical Thrombotic event	3782	144	NA	NA	NA
Ruud, E. (2006)[82]	Norway	PC	ALL	Effect of asparaginase	30	0	NA	15 asymptomatic CVC-related VTE	NA
Mohren, M. (2006)[83]	Germany	RC	Acute leukemia	Clinical Thrombotic	455	55	NA[78]	27 CVC-related VTE	NA

				event					
Kuskonmaz, B. (2006)[84]	Turkey	RC	ALL	Determine The neurologic complications in leukemia	206	1	NA	NA	NA
Khorana, A. A. (2006)[85]	USA	RC	Leukemia	Clinical Thrombotic event	14600	641	NA	NA	NA
Journeycake, J. M. (2006)[85]	USA	RC	AML	Evaluation of complication of catheter	20	0	NA	All patients inserted with CVC	NA
Jimenez-Zepeda, V. H. (2006)[86]	Mexico	RC	PCL	Clinical Thrombotic event	10	2	NA	NA	NA
Gladish, G. W. (2006)[87]	USA	RC	Leukemia	Clinical Thrombotic event	18	1	NA	NA	NA
Fiegl, M. (2006)[88]	Austria	RC	CLL	Clinical trials	115	1	NA	NA	NA
Delannoy, A. (2006)[89]	Belgium	PC	ALL	Clinical trials	30	3	NA	NA	YES
Chanan-Khan, A. (2006)[90]	USA	PC	CLL	Clinical trials	45	2	No primary thromboprophylaxis	NA	NA
Carr, E. (2006)[91]	USA	RC	ALL	Evaluation of complication of catheter	115	0	NA	All patients inserted with CVC	NA
Blom, J. W. (2006)[92]	the Netherlands	RC	Leukemia	Clinical Thrombotic event	1389	26	NA	NA	NA
Ziegler, S. (2006)[93]	Austria	RC	Acute leukemia	Clinical Thrombotic event	719	14	NA	1 CVC-related VTE	NA
White, R. H. (2005)[94]	USA	RC	Leukemia	Clinical Thrombotic event	7066	19	NA	NA	NA
Silver, R. T. (2005)[95]	USA	PC	CML	Clinical trials	38	6	NA	NA	8 BMT
Stiakaki, E. (2005)[96]	Greece	PC	ALL	Effect of Thrombophilic Traits	19	1	Thromboprophylaxis regimen for CVC	29 inserted with CVC<br>1 CVC-related VTE	NA
Schlenk, R. F. (2005)[97]	Germany	PC	APL	Clinical trials	82	1	NA	NA	Partial
Santoro, N. (2005)[98]	Italy	RC	ALL	Effect of sinovenous thrombosis ineukemia l	2318	11	NA	NA	NA
Nadir, Y. (2005)[99]	Israel	PC	Acute leukemia	Effect of hemostatic alteration	51	10	NA	NA	NA
McLean, T. W. (2005)[100]	USA	RC	ALL	Evaluation of complication of catheter	362	22	NA	All patients inserted with CVL<br>22 CVL-related VTE	NA
Massenkeil, G. (2005)[101]	Germany	RC	Acute leukemia	Evaluation of transplantation 移植	25	1	NA	NA	ALL SCT
Hussein, M. A. (2005)[102]	USA	PC	CLL	Clinical trials	49	1	NA	NA	NA
De Stefano, V. (2005)[103]	Italy	PC	ALL	Clinical Thrombotic event	379	24	No primary thromboprophylaxis	NA	NA
Dally, N.	Israel	RC	APL	Clinical	34	4	NA	NA	NA

(2005)[104]				Thrombotic event					
Chanan-Khan, A. (2005)[105]	USA	PC	CLL	Clinical trials	13	2	NA	NA	NA
Athale, U. H. (2005)[106]	Canada	PC	ALL	Clinical Thrombotic event	71	10	NA	1 CVC-related VTE	NA
Thomas, D. A. (2004)[107]	USA	PC	ALL	Clinical trials	20	2	NA	NA	10 allogeneic stem cell transplantation
Elliott, M. A. (2004)[108]	USA	RC	ALL	Clinical Thrombotic event	17<br>37	0<br>8	AT supplement<br>Treatment	98% inserted with Hickman catheter	NA
Beinart, G. (2004)[109]	USA	RC	ALL	Clinical Thrombotic event	91	5	NA	NA	NA
Raffoux, E. (2003)[110]	France	PC	APL	Clinical trials	20	2	NA	NA	NA
Nowak-Gottl, U. (2003)[111]	Germany	PC	ALL	Clinical Thrombotic event	56	1	NA	Prtial patients use of Broviac, Hickman, or Porth catheters	NA
Mitchell, L. G. (2003)[112]	Canada	PC	ALL	Clinical Thrombotic event	60	3	NA	All patients insertion with CVL	NA
Male, C. (2003)[113]	Canada	PC	ALL	Evaluation of complication of catheter	85	4	NA	All patients insertion with CVC	NA
Giordano, P. (2003)[114]	Italy	RC	ALL	Clinical Thrombotic event	2318	22	NA	10/22 has inserted CVL	NA
Nowak-Gottl, U. (2001)[115]	Germany	PC	ALL	Clinical Thrombotic event	120	1	NA	All patients insertion with CVC	NA
Elhasid, R. (2001)[116]	Israel	PC<br>RC	ALL	Effect of thromboprophyl-axis	41(prophylaxis)<br>50(Non-prophylaxis)	0<br>2	LMWH	NA	NA
Mauz-Korholz, C. (2000)[117]	Germany	PC	ALL	Effect of Thrombophilic Traits	108	3	NA	3 CVC-related VTE	NA
Dreger, P. (2000)[118]	Germany	PC	CLL	Effect of transplantation	20	1	NA	NA	All PBSC
Chiusolo, P. (2000)[119]	Italy	PC	Leukemia	Assessment of inherited prothrombotic risk fators	24	2	NA	1 CVC-related VTE	All SCT
Abshire, T. C. (2000)[120]	USA	PC	ALL	Clinical trials	148	1	NA	NA	NO
Wermes, C. (1999)[121]	Germany	PC	Acute leukemia	Evaluate of Thrombophilic Traits	84	6	NA	All patients insertion with CVC	NA
Ratcliffe, M. (1999)[122]	UK	PC	AML	Effect of thromboprophyl-axis	10	1	Low-dose warfarin	All patients insertion with CVC	NA
Nowak-Gottl, U. (1999)[123]	Germany	PC	ALL	Evaluate of Thrombophilic Traits	289	35	NA	1 CVC-related VTE	NA
Levitan, N. (1999)[124]	USA	RC	Leukemia	Evaluate of Thrombophilic Traits	47,234	591	NA	NA	NA
Knofler, R. (1999)[125]	Germany	PC	Acute leukemia	Evaluate of Thrombophilic Traits	30	4	NA	All patients insertion with CVC	NA
Bouabdallah, R. (1999)[126]	France	PC	AML	Clinical trials	51	2	NA	NA	NA
Todeschini, G. (1998)[127]	Italy	PC	ALL	Clinical trials	60	1	NA	NA	7 allogeneic BMT

Larson, R. A. (1998)[128]	USA	RC	ALL	Effect of L-asparaginase	141	8	NA	NA	NA
Chaffanjon, P. C. (1998)[129]	France	PC	CLL	Effect of Splenectomy	12	1	Administer LMWH when PLT> 30,000/ml	NA	NA
Rees, D. (1997)[130]	UK	RC	APL	Evaluate of genetic thrombophilic Traits	48	3			NA
Leoni, F. (1997)[131]	Italy	PC	AML	Clinical trials	66	1	NA	NA	NA
Uderzo, C. (1995)[132]	Italy	RC	Acute leukemia	Clinical Thrombotic event	430	17	3(DIC)/17 receiving loe-dose heparin	Partial inserted with CVC	93BMT
Uderzo, C. (1993)[133]	Italy	RC	Leukemia	Clinical Thrombotic Event undergoing BMT	67	3	NA	NA	NA
Gugliotta, L. (1992)[134]	Italy	RC	ALL	Clinical Thrombotic event	238	8	NA	NA	NA
Anger, B. R. (1989)[134]	Germany	RC	CML	Clinical Thrombotic event	232	0	NA	NA	partial
Goldberg, M. A. (1987)[134]	USA	RC	APL	Evaulate of hemostatic alteration	27	1	Comprehensive use of FFP or heparin	NA	NA
Needleman, S. W. (1981)[135]	USA	RC	Acute leukemia	Clinical Thrombotic event	80	3	NA	NA	NA
Priest, J. R. (1982)[136]	USA	RC	ALL	Effect of L-asparaginase	1，547	8	NA	NA	NA
Escudier, S. M. (1996)[137]	USA	RC	APL	Clinical Thrombotic event	56	2	NA	NA	NA
Cetkovsky, P. (1995)[138]	Czech Republic	PC	AML	Evaulate of hemostatic alteration	28	1	NA	1 CVC-related VTE	NA
Nowak-Goettl, U. (1994)[139]	Germany	PC	ALL	Evaulate of hemostatic alteration	46	4	NA	4 CVC-related VTE	NA
Cortes, J. E. (1994)[140]	USA	PC	APL	Clinical trials	17	1	No herapin prophylaxis	NA	Partial
Mitchell, L. (1994)[141]	Canada	PC	ALL	Evaulate of hemostatic alteration	26	3	NA	NA	NA
Sarris, A. H. (1992)[142]	USA	RC	ALL	Evaulate of hemostatic alteration	153	4	NA	NA	NA
Blatt, J. (1989)[143]	USA	RC	ALL	Evaulate of hemostatic alteration	217	1	NA	NA	NA
Zaunschirm, A. (1986)[144]	Austria	RC	ALL	Effect of thromboprophylaxis	13	0	AT concentrate	NA	NA

VTE=venous thromboembolism.DVT=deep venous thrombosis. PC=prospective cohort study. RC=retrospective cohort study. PICC=peripherally inserted central catheter. CVL=central venous line. ALL=acute lymphoblastic leukemia. CLL=chronic lymphoblastic leukemia. AML= acute myeloid leukemia. CML= chronic myeloid leukemia. APL= acute promyelocytic leukemia. PPCL= primary plasma cell leukemia. CML-CP=chronic myelogenous leukemia in chronic phase.HCT= hematopoietic cell transplantation.SCT= stem cell transplantation. BMT= bone marrow transplantation.FFP= fresh-frozen plasma. LMWH= low molecular weight heparin.AT=antithrombin.DIC= disseminated intravascular coagulation.PLT=platelet.

Reference

1. Lee, Y.G. et al. Implications of cytogenetics for venous thromboembolism in acute myeloid leukaemia. *Thrombosis and haemostasis* 113, 201-208 (2015).
2. Ranta, S. et al. Cerebral sinus venous thromboses in children with acute lymphoblastic leukaemia - a multicentre study from the Nordic Society of Paediatric Haematology and Oncology. *British journal of haematology* (2014).
3. Santos, F.P. et al. Splenectomy in patients with myeloproliferative neoplasms: efficacy, complications and impact on survival and transformation. *Leukemia & Lymphoma* 55, 121-127.
4. O'Hara, V.J., Miller, T., Mehta, R., Swartzendruber, E. & Kiel, P.J. Incidence of venous thromboembolism in the setting of hematopoietic cell transplantation. *American Journal of*

Therapeutics 21, 15-19.
5. Musto, P. et al. Lenalidomide and low-dose dexamethasone for newly diagnosed primary plasma cell leukemia. *Leukemia* 28, 222-225.
6. Morano, S.G. et al. Early and late complications related to central venous catheters in hematological malignancies: a retrospective analysis of 1102 patients. *Mediterranean journal of hematology and infectious diseases* 6, e2014011 (2014).
7. Mitrovic, M. et al. Thrombotic events in acute promyelocytic leukemia. *Thrombosis research* (2014).
8. James, D.F. et al. Lenalidomide and rituximab for the initial treatment of patients with chronic lymphocytic leukemia: a multicenter clinical-translational study from the chronic lymphocytic leukemia research consortium. *Journal of Clinical Oncology* 32, 2067-2073.
9. Bach, A.G. et al. Pulmonary embolism in oncologic patients: frequency and embolus burden of symptomatic and unsuspected events. *Acta Radiologica* 55, 45-53.
10. Avila, M.L. et al. Assessment of the outcomes associated with periprocedural anticoagulation management in children with acute lymphoblastic leukemia. *The Journal of pediatrics* 164, 1201-1207 (2014).
11. Abraham, A. et al. Outcome of treatment with a low cost protocol in adults with T cell acute lymphoblastic leukemia in a tertiary care center in India. *Leukemia & Lymphoma* 55, 947-949.
12. Walker, A.J., Card, T.R., West, J., Crooks, C. & Grainge, M.J. Incidence of venous thromboembolism in patients with cancer - a cohort study using linked United Kingdom databases. *European Journal of Cancer* 49, 1404-1413.
13. Vrooman, L.M. et al. Postinduction dexamethasone and individualized dosing of Escherichia Coli L-asparaginase each improve outcome of children and adolescents with newly diagnosed acute lymphoblastic leukemia: results from a randomized study--Dana-Farber Cancer Institute ALL Consortium Protocol 00-01. *Journal of clinical oncology : official journal of the American Society of Clinical Oncology* 31, 1202-1210 (2013).
14. Strati, P. et al. Lenalidomide induces long-lasting responses in elderly patients with chronic lymphocytic leukemia. *Blood* 122, 734-737.
15. Santoro, N. et al. Screening for coagulopathy and identification of children with acute lymphoblastic leukemia at a higher risk of symptomatic venous thrombosis: an AIEOP experience. *Journal of pediatric hematology/oncology* 35, 348-355 (2013).
16. Ranta, S. et al. Antithrombin deficiency after prolonged asparaginase treatment in children with acute lymphoblastic leukemia. *Blood coagulation & fibrinolysis : an international journal in haemostasis and thrombosis* 24, 749-756 (2013).
17. Lim, M.Y. et al. Comparison of complication rates of Hickman((R)) catheters versus peripherally inserted central catheters in patients with acute myeloid leukemia undergoing induction chemotherapy. *Leukemia & lymphoma* 54, 1263-1267 (2013).
18. Lauw, M.N. et al. Venous thromboembolism in adults treated for acute lymphoblastic leukaemia: Effect of fresh frozen plasma supplementation. *Thrombosis and haemostasis* 109, 633-642 (2013).
19. Guzman-Uribe, P., Rosas-Lopez, A., Zepeda-Leon, J. & Crespo-Solis, E. Incidence of thrombosis in adults with acute leukemia: a single center experience in Mexico. *Revista de investigacion clinica; organo del Hospital de Enfermedades de la Nutricion* 65, 130-140 (2013).
20. Grisariu, S., Spectre, G., Kalish, Y. & Gatt, M.E. Increased risk of central venous catheter-associated thrombosis in acute promyelocytic leukemia: a single-institution experience. *European journal of haematology* 90, 397-403 (2013).
21. Chang, H. et al. Acute promyelocytic leukemia-associated thrombosis. *Acta haematologica* 130, 1-6 (2013).
22. Albisetti, M. et al. Port-a-cath-related thrombosis and postthrombotic syndrome in pediatric oncology patients. *The Journal of pediatrics* 163, 1340-1346 (2013).
23. Vos, F.J. et al. 18F-FDG PET/CT for diagnosing infectious complications in patients with severe neutropenia after intensive chemotherapy for haematological malignancy or stem cell transplantation. *European journal of nuclear medicine and molecular imaging* 39, 120-128 (2012).
24. Torun, Y.A. et al. Inherited prothrombotic risk factors in Turkish children with acute lymphoblastic leukemia: significance of concomitant genetic mutation. *Clinical and applied thrombosis/hemostasis : official journal of the International Academy of Clinical and Applied Thrombosis/Hemostasis* 18, 218-221 (2012).
25. Thyagu, S. et al. Treatment of Philadelphia chromosome-positive acute lymphoblastic leukaemia with imatinib combined with a paediatric-based protocol. *British Journal of Haematology* 158, 506-514.
26. Rollig, C. et al. Survey and analysis of the efficacy and prescription pattern of sorafenib in patients with acute myeloid leukemia. *Leukemia & Lymphoma* 53, 1062-1067.
27. Pollyea, D.A. et al. Safety, efficacy and biological predictors of response to sequential azacitidine and lenalidomide for elderly patients with acute myeloid leukemia. *Leukemia* 26, 893-901.
28. Pettitt, A.R. et al. Alemtuzumab in combination with methylprednisolone is a highly effective induction regimen for patients with chronic lymphocytic leukemia and deletion of TP53: final results of the national cancer research institute CLL206 trial. *Journal of Clinical Oncology* 30, 1647-1655.
29. Lejhancova-Tousovska, K., Zapletal, O., Vytiskova, S., Strbackova, P. & Sterba, J. Profile of thrombin generation in children with acute lymphoblastic leukemia treated by Berlin-Frankfurt-Munster (BFM) protocols. *Blood coagulation & fibrinolysis : an international journal in haemostasis and thrombosis* 23, 144-154 (2012).
30. Gonzalez, G. et al. Safety of central venous catheter placement at diagnosis of acute lymphoblastic leukemia in children. *Pediatric blood & cancer* 58, 498-502 (2012).
31. Akin, D.F. et al. Factor V Leiden and Prothrombin 20210A Mutations among Turkish Pediatric Leukemia Patients. *Leukemia research and treatment* 2012, 250432 (2012).
32. Whittle, A.M., Allsup, D.J. & Bailey, J.R. Chronic lymphocytic leukaemia is a risk factor for venous thromboembolism. *Leukemia research* 35, 419-421 (2011).
33. Topp, M.S. et al. Targeted therapy with the T-cell-engaging antibody blinatumomab of chemotherapy-refractory minimal residual disease in B-lineage acute lymphoblastic leukemia patients results in high response rate and prolonged leukemia-free survival. *Journal of clinical oncology : official journal of the American Society of Clinical Oncology* 29, 2493-2498 (2011).
34. Santos, F.P. et al. Long-term prognostic impact of the use of erythropoietic-stimulating agents in patients with chronic myeloid leukemia in chronic phase treated with imatinib. *Cancer* 117, 982-991 (2011).
35. O'Brien, S.H., Klima, J., Termuhlen, A.M. & Kelleher, K.J. Venous thromboembolism and adolescent and young adult oncology inpatients in US children's hospitals, 2001 to 2008. *The Journal of pediatrics* 159, 133-137 (2011).
36. Montillo, M. et al. An open-label, pilot study of fludarabine, cyclophosphamide, and alemtuzumab in relapsed/refractory patients with B-cell chronic lymphocytic leukemia. *Blood* 118, 4079-4085.
37. Lipay, N.V., Zmitrovich, A.I. & Aleinikova, O.V. Epidemiology of venous thromboembolism in children with malignant diseases: A single-center study of the Belarusian Center for Pediatric Oncology and Hematology. *Thrombosis research* 128, 130-134 (2011).
38. Latagliata, R. et al. Dasatinib is safe and effective in unselected chronic myeloid leukaemia elderly patients resistant/intolerant to imatinib. *Leukemia Research* 35, 1164-1169.
39. Hunault-Berger, M. et al. A randomized study of pegylated liposomal doxorubicin versus continuous-infusion doxorubicin in elderly patients with acute lymphoblastic leukemia: the GRAALL-SA1 study. *Haematologica* 96, 245-252.
40. Grace, R.F. et al. The frequency and management of asparaginase-related thrombosis in paediatric and adult patients with acute lymphoblastic leukaemia treated on Dana-Farber Cancer Institute consortium protocols. *British journal of haematology* 152, 452-459 (2011).
41. Giordano, P. et al. Magnetic resonance imaging screening of cerebral thromboembolic events in children with acute lymphoblastic leukemia: a pilot study. *Neuropediatrics* 42, 55-59 (2011).
42. Freyer, D.R. et al. Postrelapse survival in childhood acute lymphoblastic leukemia is independent of initial treatment intensity: a report from the Children's Oncology Group. *Blood* 117,

3010-3015.

43. Faderl, S. et al. Augmented hyper-CVAD based on dose-intensified vincristine, dexamethasone, and asparaginase in adult acute lymphoblastic leukemia salvage therapy. *Clinical lymphoma, myeloma & leukemia* 11, 54-59.

44. Bezares, R.F. et al. Multicenter study of subcutaneous alemtuzumab administered at reduced dose in patients with fludarabine-relapsed/refractory chronic lymphocytic leukemia: final analysis. *Leukemia & Lymphoma* 52, 1936-1941.

45. Aue, G. et al. Inflammation, TNFalpha and endothelial dysfunction link lenalidomide to venous thrombosis in chronic lymphocytic leukemia. *American journal of hematology* 86, 835-840 (2011).

46. Al-Aridi, C. et al. Thrombosis in children with acute lymphoblastic leukemia treated at a tertiary care center in Lebanon: revisiting the role of predictive models. *Pediatric hematology and oncology* 28, 676-681 (2011).

47. Visani, G. et al. Dasatinib, even at low doses, is an effective second-line therapy for chronic myeloid leukemia patients resistant or intolerant to imatinib. Results from a real life-based Italian multicenter retrospective study on 114 patients. *American Journal of Hematology* 85, 960-963.

48. Tran, H. et al. Deep venous thromboses in patients with hematological malignancies after peripherally inserted central venous catheters. *Leukemia & Lymphoma* 51, 1473-1477.

49. Silverman, L.B. et al. Intravenous PEG-asparaginase during remission induction in children and adolescents with newly diagnosed acute lymphoblastic leukemia. *Blood* 115, 1351-1353 (2010).

50. Ribera, J.M. et al. Concurrent intensive chemotherapy and imatinib before and after stem cell transplantation in newly diagnosed Philadelphia chromosome-positive acute lymphoblastic leukemia. Final results of the CSTIBES02 trial. *Haematologica* 95, 87-95.

51. Revel-Vilk, S. et al. Risk factors for central venous catheter thrombotic complications in children and adolescents with cancer. *Cancer* 116, 4197-4205.

52. Ravandi, F. et al. First report of phase 2 study of dasatinib with hyper-CVAD for the frontline treatment of patients with Philadelphia chromosome-positive (Ph+) acute lymphoblastic leukemia. *Blood* 116, 2070-2077.

53. Qureshi, A., Mitchell, C., Richards, S., Vora, A. & Goulden, N. Asparaginase-related venous thrombosis in UKALL 2003-re-exposure to asparaginase is feasible and safe. *British journal of haematology* 149, 410-413 (2010).

54. Peris, A. et al. Implantation of 3951 long-term central venous catheters: performances, risk analysis, and patient comfort after ultrasound-guidance introduction. *Anesthesia and analgesia* 111, 1194-1201 (2010).

55. Ociepa, T., Maloney, E., Urasinski, T. & Sawicki, M. Thrombotic complications of tunneled central lines in children with malignancy. *Journal of Pediatric Hematology/Oncology* 32, 88-92.

56. Mitchell, L. et al. Validation of a predictive model for identifying an increased risk for thromboembolism in children with acute lymphoblastic leukemia: results of a multicenter cohort study. *Blood* 115, 4999-5004 (2010).

57. Leupin, N. et al. Efficacy of rituximab and cladribine in patients with chronic lymphocytic leukemia and feasibility of stem cell mobilization: a prospective multicenter phase II trial (protocol SAKK 34/02). *Leukemia & Lymphoma* 51, 613-619.

58. Harlev, D. et al. Prophylactic therapy with enoxaparin in children with acute lymphoblastic leukemia and inherited thrombophilia during L-asparaginase treatment. *Thrombosis research* 126, 93-97 (2010).

59. Giordano, P. et al. Prospective study of hemostatic alterations in children with acute lymphoblastic leukemia. *American journal of hematology* 85, 325-330 (2010).

60. Storring, J.M. et al. Treatment of adults with BCR-ABL negative acute lymphoblastic leukaemia with a modified paediatric regimen. *British journal of haematology* 146, 76-85 (2009).

61. Blum, W. et al. Dose escalation of lenalidomide in relapsed or refractory acute leukemias. *Journal of Clinical Oncology* 28, 4919-4925.

62. Pinon, M. et al. A prospective 7-year survey on central venous catheter-related complications at a single pediatric hospital. *European journal of pediatrics* 168, 1505-1512 (2009).

63. Ku, G.H. et al. Venous thromboembolism in patients with acute leukemia: incidence, risk factors, and effect on survival. *Blood* 113, 3911-3917 (2009).

64. Eloraby, A.M. L-asparaginase therapy with concomitant cranial venous thrombosis: can MRI help avoiding stroke. *Journal of the Egyptian National Cancer Institute* 21, 43-50 (2009).

65. Castro, J.E. et al. Rituximab in combination with high-dose methylprednisolone for the treatment of chronic lymphocytic leukemia. *Leukemia* 23, 1779-1789.

66. Blum, K.A. et al. Phase II study of the histone deacetylase inhibitor MGCD0103 in patients with previously treated chronic lymphocytic leukaemia. *British Journal of Haematology* 147, 507-514.

67. Abbott, L.S. et al. The impact of prophylactic fresh-frozen plasma and cryoprecipitate on the incidence of central nervous system thrombosis and hemorrhage in children with acute lymphoblastic leukemia receiving asparaginase. *Blood* 114, 5146-5151 (2009).

68. Wassenaar, T. et al. Acute promyelocytic leukaemia and acquired alpha-2-plasmin inhibitor deficiency: a retrospective look at the use of epsilon-aminocaproic acid (Amicar) in 30 patients. *Hematological oncology* 26, 241-246 (2008).

69. Pieters, R. et al. Pharmacokinetics, pharmacodynamics, efficacy, and safety of a new recombinant asparaginase preparation in children with previously untreated acute lymphoblastic leukemia: a randomized phase 2 clinical trial. *Blood* 112, 4832-4838.

70. Meister, B. et al. Comparison of low-molecular-weight heparin and antithrombin versus antithrombin alone for the prevention of symptomatic venous thromboembolism in children with acute lymphoblastic leukemia. *Pediatric blood & cancer* 50, 298-303 (2008).

71. Hess, G. et al. Sustained remissions and low rate of BCR-ABL resistance mutations with imatinib treatment chronic myelogenous leukemia in patients treated in late chronic phase: a 5-year follow up. *American Journal of Hematology* 83, 178-184.

72. Ferrajoli, A. et al. Lenalidomide induces complete and partial remissions in patients with relapsed and refractory chronic lymphocytic leukemia. *Blood* 111, 5291-5297.

73. Ottmann, O.G. et al. Imatinib compared with chemotherapy as front-line treatment of elderly patients with Philadelphia chromosome-positive acute lymphoblastic leukemia (Ph+ALL). *Cancer* 109, 2068-2076.

74. Melillo, L. et al. Symptomatic venous thromboembolism and thrombophilic status in adult acute leukemia: a single-center experience of 114 patients at diagnosis. *Acta haematologica* 117, 215-220 (2007).

75. Farinasso, L. et al. Risk factors of central venous lines-related thrombosis in children with acute lymphoblastic leukemia during induction therapy: a prospective study. *Leukemia* 21, 552-556 (2007).

76. Khorana, A.A., Francis, C.W., Culakova, E., Kuderer, N.M. & Lyman, G.H. Frequency, risk factors, and trends for venous thromboembolism among hospitalized cancer patients. *Cancer* 110, 2339-2346 (2007).

77. Douer, D. et al. Pharmacodynamics and safety of intravenous pegaspargase during remission induction in adults aged 55 years or younger with newly diagnosed acute lymphoblastic leukemia. *Blood* 109, 2744-2750.

78. Breccia, M. et al. Occurrence of thrombotic events in acute promyelocytic leukemia correlates with consistent immunophenotypic and molecular features. *Leukemia* 21, 79-83 (2007).

79. Barr, P. et al. Antiangiogenic activity of thalidomide in combination with fludarabine, carboplatin, and topotecan for high-risk acute myelogenous leukemia. *Leukemia & lymphoma* 48, 1940-1949 (2007).

80. Thomas, D.A. et al. Phase II study of sphingosomal vincristine in patients with recurrent or refractory adult acute lymphocytic leukemia. *Cancer* 106, 120-127.

81. Sakuma, M. et al. Cancer and pulmonary embolism: thrombotic embolism, tumor embolism, and tumor invasion into a large vein. *Circulation journal : official journal of the Japanese Circulation Society* 70, 744-749 (2006).

82. Ruud, E. et al. Thrombotic effects of asparaginase in two acute lymphoblastic leukemia protocols (NOPHO ALL-1992 versus NOPHO ALL-2000): a single-institution study. *Pediatric hematology and oncology* 23, 207-216 (2006).
83. Mohren, M. et al. Increased risk of venous thromboembolism in patients with acute leukaemia. *British journal of cancer* 94, 200-202 (2006).
84. Kuskonmaz, B. et al. The neurologic complications in pediatric acute lymphoblastic leukemia patients excluding leukemic infiltration. *Leukemia research* 30, 537-541 (2006).
85. Khorana, A.A. et al. Thromboembolism in hospitalized neutropenic cancer patients. *Journal of clinical oncology : official journal of the American Society of Clinical Oncology* 24, 484-490 (2006).
86. Jimenez-Zepeda, V.H. & Dominguez, V.J. Plasma cell leukemia: A rare condition. *Annals of Hematology* 85, 263-267 (2006).
87. Gladish, G.W. et al. Incidental pulmonary emboli in oncology patients: prevalence, CT evaluation, and natural history. *Radiology* 240, 246-255 (2006).
88. Fiegl, M. et al. Routine clinical use of alemtuzumab in patients with heavily pretreated B-cell chronic lymphocytic leukemia: a nation-wide retrospective study in Austria. *Cancer* 107, 2408-2416.
89. Delannoy, A. et al. Imatinib and methylprednisolone alternated with chemotherapy improve the outcome of elderly patients with Philadelphia-positive acute lymphoblastic leukemia: results of the GRAALL AFR09 study. *Leukemia* 20, 1526-1532.
90. Chanan-Khan, A. et al. Clinical efficacy of lenalidomide in patients with relapsed or refractory chronic lymphocytic leukemia: results of a phase II study. *Journal of Clinical Oncology* 24, 5343-5349.
91. Carr, E. et al. The safety of central line placement prior to treatment of pediatric acute lymphoblastic leukemia. *Pediatric blood & cancer* 47, 886-888 (2006).
92. Blom, J.W. et al. Incidence of venous thrombosis in a large cohort of 66,329 cancer patients: results of a record linkage study. *Journal of thrombosis and haemostasis : JTH* 4, 529-535 (2006).
93. Ziegler, S. et al. Symptomatic venous thromboembolism in acute leukemia. Incidence, risk factors, and impact on prognosis. *Thrombosis research* 115, 59-64 (2005).
94. White, R.H. et al. Incidence of venous thromboembolism in the year before the diagnosis of cancer in 528,693 adults. *Archives of internal medicine* 165, 1782-1787 (2005).
95. Silver, R.T. Anagrelide is effective in treating patients with hydroxyurea-resistant thrombocytosis in patients with chronic myeloid leukemia. *Leukemia* 19, 39-43 (2005).
96. Stiakaki, E. et al. Prevalence of Factor V Leiden and other thrombophilic traits among Cretan children with malignancy. *Pediatric blood & cancer* 44, 386-389 (2005).
97. Schlenk, R.F. et al. High-dose cytarabine and mitoxantrone in consolidation therapy for acute promyelocytic leukemia. *Leukemia* 19, 978-983.
98. Santoro, N. et al. Ischemic stroke in children treated for acute lymphoblastic leukemia: a retrospective study. *Journal of pediatric hematology/oncology* 27, 153-157 (2005).
99. Nadir, Y. et al. Hemostatic balance on the surface of leukemic cells: the role of tissue factor and urokinase plasminogen activator receptor. *Haematologica* 90, 1549-1556 (2005).
100. McLean, T.W., Fisher, C.J., Snively, B.M. & Chauvenet, A.R. Central venous lines in children with lesser risk acute lymphoblastic leukemia: optimal type and timing of placement. *Journal of clinical oncology : official journal of the American Society of Clinical Oncology* 23, 3024-3029 (2005).
101. Massenkeil, G. et al. Survival after reduced-intensity conditioning is not inferior to standard high-dose conditioning before allogeneic haematopoietic cell transplantation in acute leukaemias. *Bone Marrow Transplantation* 36, 683-689.
102. Hussein, M.A. et al. Cyclophosphamide followed by fludarabine for untreated chronic lymphocytic leukemia: a phase II SWOG TRIAL 9706. *Leukemia* 19, 1880-1886.
103. De Stefano, V. et al. The risk of thrombosis in patients with acute leukemia: occurrence of thrombosis at diagnosis and during treatment. *Journal of thrombosis and haemostasis : JTH* 3, 1985-1992 (2005).
104. Dally, N. et al. Predictive factors of bleeding and thrombosis during induction therapy in acute promyelocytic leukemia-a single center experience in 34 patients. *Thrombosis research* 116, 109-114 (2005).
105. Chanan-Khan, A. et al. Results of a phase 1 clinical trial of thalidomide in combination with fludarabine as initial therapy for patients with treatment-requiring chronic lymphocytic leukemia (CLL). *Blood* 106, 3348-3352.
106. Athale, U.H., Siciliano, S.A., Crowther, M., Barr, R.D. & Chan, A.K. Thromboembolism in children with acute lymphoblastic leukaemia treated on Dana-Farber Cancer Institute protocols: effect of age and risk stratification of disease. *British journal of haematology* 129, 803-810 (2005).
107. Thomas, D.A. et al. Treatment of Philadelphia chromosome-positive acute lymphocytic leukemia with hyper-CVAD and imatinib mesylate. *Blood* 103, 4396-4407.
108. Elliott, M.A. et al. Thromboembolism in adults with acute lymphoblastic leukemia during induction with L-asparaginase-containing multi-agent regimens: incidence, risk factors, and possible role of antithrombin. *Leukemia & lymphoma* 45, 1545-1549 (2004).
109. Beinart, G. & Damon, L. Thrombosis associated with L-asparaginase therapy and low fibrinogen levels in adult acute lymphoblastic leukemia. *American journal of hematology* 77, 331-335 (2004).
110. Raffoux, E. et al. Combined treatment with arsenic trioxide and all-trans-retinoic acid in patients with relapsed acute promyelocytic leukemia. *Journal of Clinical Oncology* 21, 2326-2334.
111. Nowak-Gottl, U. et al. Thromboembolic events in children with acute lymphoblastic leukemia (BFM protocols): prednisone versus dexamethasone administration. *Blood* 101, 2529-2533 (2003).
112. Mitchell, L.G. et al. A prospective cohort study determining the prevalence of thrombotic events in children with acute lymphoblastic leukemia and a central venous line who are treated with L-asparaginase: results of the Prophylactic Antithrombin Replacement in Kids with Acute Lymphoblastic Leukemia Treated with Asparaginase (PARKAA) Study. *Cancer* 97, 508-516 (2003).
113. Male, C. et al. Central venous line-related thrombosis in children: association with central venous line location and insertion technique. *Blood* 101, 4273-4278 (2003).
114. Giordano, P. et al. T-immunophenotype is associated with an increased prevalence of thrombosis in children with acute lymphoblastic leukemia. A retrospective study. *Haematologica* 88, 1079-1080.
115. Nowak-Gottl, U. et al. Thrombotic events revisited in children with acute lymphoblastic leukemia: impact of concomitant Escherichia coli asparaginase/prednisone administration. *Thrombosis research* 103, 165-172 (2001).
116. Elhasid, R. et al. Prophylactic therapy with enoxaparin during L-asparaginase treatment in children with acute lymphoblastic leukemia. *Blood coagulation & fibrinolysis : an international journal in haemostasis and thrombosis* 12, 367-370 (2001).
117. Mauz-Korholz, C., Junker, R., Gobel, U. & Nowak-Gottl, U. Prothrombotic risk factors in children with acute lymphoblastic leukemia treated with delayed E. coli asparaginase (COALL-92 and 97 protocols). *Thrombosis and haemostasis* 83, 840-843 (2000).
118. Dreger, P. et al. A prospective study of positive/negative ex vivo B-cell depletion in patients with chronic lymphocytic leukemia. *Experimental hematology* 28, 1187-1196 (2000).
119. Chiusolo, P. et al. Incidence of Factor V Leiden and prothrombin G20210A in patients submitted to stem cell transplantation. *Haematologica* 85, 670-671.
120. Abshire, T.C., Pollock, B.H., Billett, A.L., Bradley, P. & Buchanan, G.R. Weekly polyethylene glycol conjugated L-asparaginase compared with biweekly dosing produces superior induction remission rates in childhood relapsed acute lymphoblastic leukemia: a Pediatric Oncology Group Study. *Blood* 96, 1709-1715 (2000).
121. Wermes, C. et al. Clinical relevance of genetic risk factors for thrombosis in paediatric oncology patients with central venous catheters. *European journal of pediatrics* 158 Suppl 3, S143-146 (1999).
122. Ratcliffe, M., Broadfoot, C., Davidson, M., Kelly, K.F. & Greaves, M. Thrombosis, markers of thrombotic risk, indwelling central venous catheters and antithrombotic prophylaxis using low-dose warfarin in subjects with malignant disease. *Clinical and laboratory haematology* 21, 353-357 (1999).
123. Nowak-Gottl, U. et al. Prospective evaluation of the thrombotic risk in children with acute lymphoblastic leukemia carrying the MTHFR TT 677 genotype, the prothrombin G20210A

variant, and further prothrombotic risk factors. *Blood* 93, 1595-1599 (1999).
124. Levitan, N. et al. Rates of initial and recurrent thromboembolic disease among patients with malignancy versus those without malignancy. Risk analysis using Medicare claims data. *Medicine* 78, 285-291 (1999).
125. Knofler, R. et al. Clinical importance of prothrombotic risk factors in pediatric patients with malignancy--impact of central venous lines. *European journal of pediatrics* 158 Suppl 3, S147-150 (1999).
126. Bouabdallah, R. et al. A phase II trial of induction and consolidation therapy of acute myeloid leukemia with weekly oral idarubicin alone in poor risk elderly patients. *Leukemia* 13, 1491-1496 (1999).
127. Todeschini, G. et al. Estimated 6-year event-free survival of 55% in 60 consecutive adult acute lymphoblastic leukemia patients treated with an intensive phase II protocol based on high induction dose of daunorubicin. *Leukemia* 12, 144-149.
128. Larson, R.A., Fretzin, M.H., Dodge, R.K. & Schiffer, C.A. Hypersensitivity reactions to L-asparaginase do not impact on the remission duration of adults with acute lymphoblastic leukemia. *Leukemia* 12, 660-665 (1998).
129. Chaffanjon, P.C., Brichon, P.Y., Ranchoup, Y., Gressin, R. & Sotto, J.J. Portal vein thrombosis following splenectomy for hematologic disease: prospective study with Doppler color flow imaging. *World journal of surgery* 22, 1082-1086 (1998).
130. Rees, D., Grimwade, D., Langabeer, S., Burnett, A. & Goldstone, A. Influence of genetic predisposition to thrombosis on natural history of acute promyelocytic leukaemia. MRC Adult Leukaemia Working Party. *British journal of haematology* 96, 490-492 (1997).
131. Leoni, F. et al. Idarubicin induction treatment of acute myeloid leukemia in the elderly. *Haematologica* 82, 13-18 (1997).
132. Uderzo, C. et al. Pulmonary thromboembolism in childhood leukemia: 8-years' experience in a pediatric hematology center. *Journal of clinical oncology : official journal of the American Society of Clinical Oncology* 13, 2805-2812 (1995).
133. Uderzo, C. et al. Pulmonary thromboembolism in leukaemic children undergoing bone marrow transplantation. *Bone marrow transplantation* 11, 201-203 (1993).
134. Gugliotta, L. et al. Incidence of thrombotic complications in adult patients with acute lymphoblastic leukaemia receiving L-asparaginase during induction therapy: a retrospective study. The GIMEMA Group. *European journal of haematology* 49, 63-66 (1992).
135. Needleman, S.W., Stein, M.N. & Hoak, J.C. Pulmonary embolism in patients with acute leukemia and severe thrombocytopenia. *The Western journal of medicine* 135, 9-13 (1981).
136. Priest, J.R. et al. A syndrome of thrombosis and hemorrhage complicating L-asparaginase therapy for childhood acute lymphoblastic leukemia. *The Journal of pediatrics* 100, 984-989 (1982).
137. Escudier, S.M., Kantarjian, H.M. & Estey, E.H. Thrombosis in patients with acute promyelocytic leukemia treated with and without all-trans retinoic acid. *Leukemia & lymphoma* 20, 435-439 (1996).
138. Cetkovsky, P., Koza, V., Cepelak, V. & Vit, L. Haemostasis in patients with acute myeloid leukaemia treated with intermediate dose of cytosine arabinoside and mitoxantrone: The influence of chemotherapy, infection and remission status on haemostasis. *Fibrinolysis* 9, 165-169 (1995).
139. Nowak-Goettl, U. et al. Enhanced thrombin generation, von Willebrand factor, fibrin D-dimer and plasminogen activator inhibitor 1: Predictive for venous thrombosis in asparaginase-treated children. *Fibrinolysis* 8, 63-65 (1994).
140. Cortes, J.E. et al. All-trans retinoic acid followed by chemotherapy for salvage of refractory or relapsed acute promyelocytic leukemia. *Cancer* 73, 2946-2952 (1994).
141. Mitchell, L., Hoogendoorn, H., Giles, A.R., Vegh, P. & Andrew, M. Increased endogenous thrombin generation in children with acute lymphoblastic leukemia: risk of thrombotic complications in L'Asparaginase-induced antithrombin III deficiency. *Blood* 83, 386-391 (1994).
142. Sarris, A.H. et al. High incidence of disseminated intravascular coagulation during remission induction of adult patients with acute lymphoblastic leukemia. *Blood* 79, 1305-1310 (1992).
143. Blatt, J., Penchansky, L. & Horn, M. Thrombocytosis as a presenting feature of acute lymphoblastic leukemia in childhood. *American journal of hematology* 31, 46-49 (1989).
144. Zaunschirm, A. & Muntean, W. Correction of hemostatic imbalances induced by L-asparaginase therapy in children with acute lymphoblastic leukemia. *Pediatric hematology and oncology* 3, 19-25 (1986).
